# External fixation of paediatric subtrochanteric fractures using calcar rather than neck pins

**DOI:** 10.1007/s11751-016-0252-8

**Published:** 2016-04-12

**Authors:** Sherif Galal

**Affiliations:** Department of Orthopaedic Surgery and Traumatology, Faculty of Medicine, Kasr AL-Ainy School of Medicine, Cairo University, Cairo, 11559 Egypt

**Keywords:** External fixator, Paediatric, Subtrochanteric, Fracture

## Abstract

Subtrochanteric femoral fractures in children are uncommon and have received limited attention in the literature. Its treatment is controversial, and different options are available: traction, spica casting, internal fixation and external fixation. The aim of this study is to present our results with external fixation of subtrochanteric femoral fractures in children using Ilizarov frame. Between January 2012 and January 2014, 14 patients with closed subtrochanteric femoral fractures were treated in Cairo University School of Medicine Teaching Hospital. The average age at the time of injury was 6.4 years (range 3.8–11.5 years). Pathological fractures and fractures associated with neuromuscular diseases were excluded from this study. Two patients were multiply injured with abdominal injuries (as ruptured spleen). In all cases, a low profile Ilizarov frame was inserted using two half pins inserted proximally from greater to lesser trochanters parallel to the hip joint orientation line (line between tip of greater trochanter and femoral head centre) and secured to an arch, and another three half pins were inserted distally perpendicular to the femoral shaft and secured to an arch that was connected by three rods to the proximal arch. No post-operative spica was used. Average follow-up was 18 months (range 12–36 months). All fractures united with anatomical alignment within an average of 8 weeks (range 6–12 weeks). There were no deep infections and no significant limb length discrepancies. At the latest follow-up, no patient had any restriction of activities. External fixation with a low profile Ilizarov frame appears as a good treatment option for subtrochanteric femoral fractures in children.

Level of evidence: Level IV.

## Introduction

Subtrochanteric femoral fractures in children are a special type starting 1–2 cm below the lesser trochanter [1]. There is difficulty maintaining fracture reduction due to the strong deforming muscle forces displacing the proximal fragment into a flexed, abducted and externally rotated position [1, 8, 9]. These forces make it difficult to maintain reduction using traction or spica casting [3]. There are increasing reports in the literature, suggesting operative treatment leads to better results than non-operative methods [13]. Methods of fixation include intramedullary nails, compression plating and external fixation [2, 10].

The aim of the study was to evaluate the results of a new configuration of the Ilizarov fixator for stabilising subtrochanteric femoral fractures in children.

## Patients and methods

**Fig. 1:**
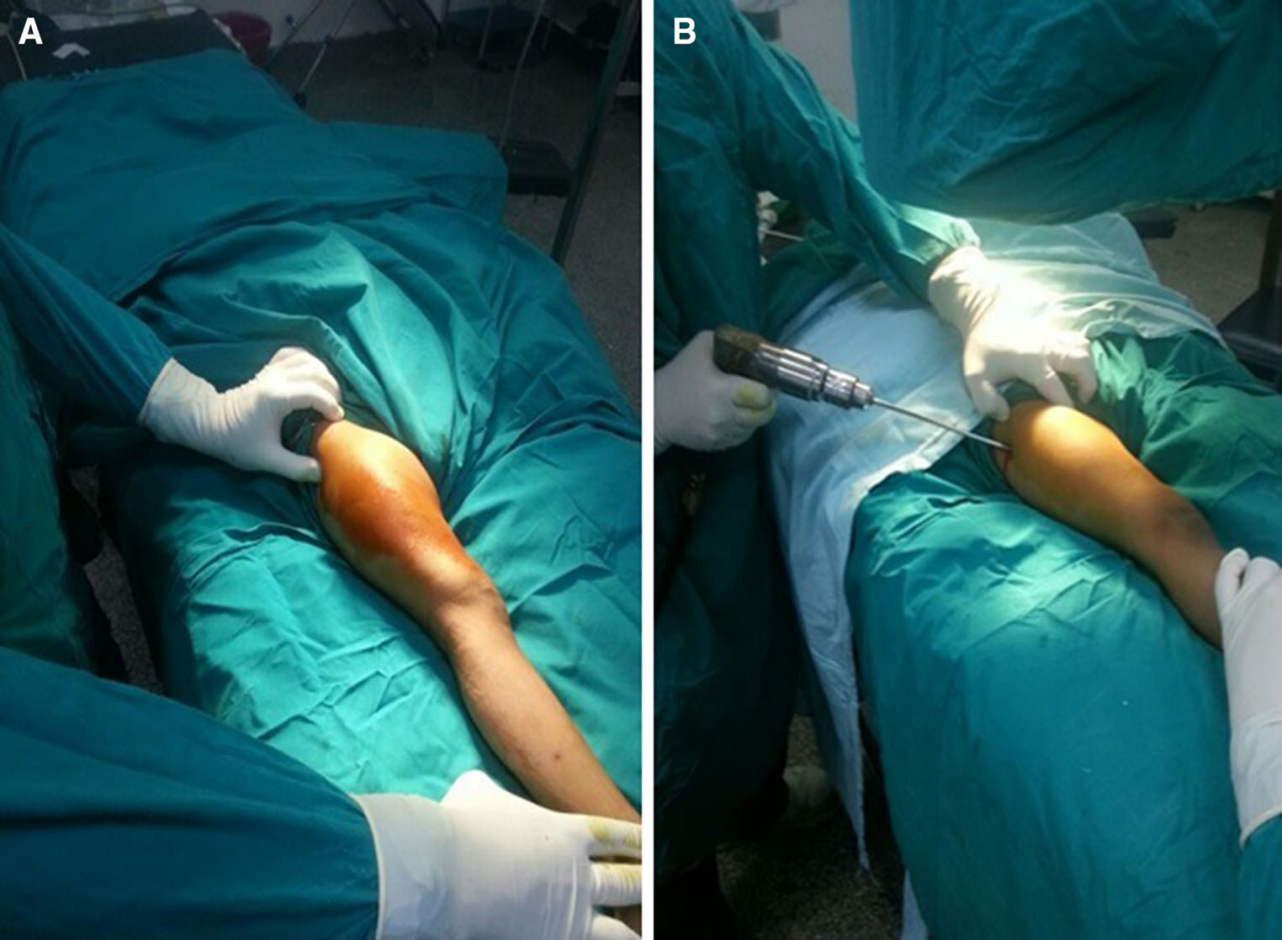
**a** Hip neutral position with leg internally rotated; **b** first proximal half pin introduced

**Fig. 2:**
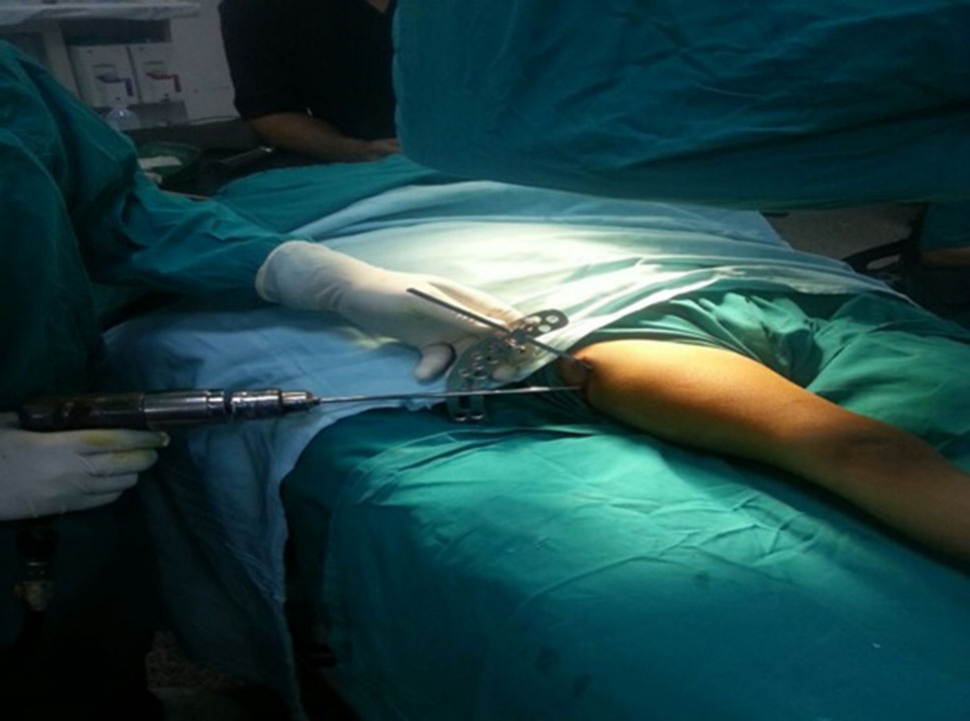
Second proximal half pin introduced

**Fig. 3:**
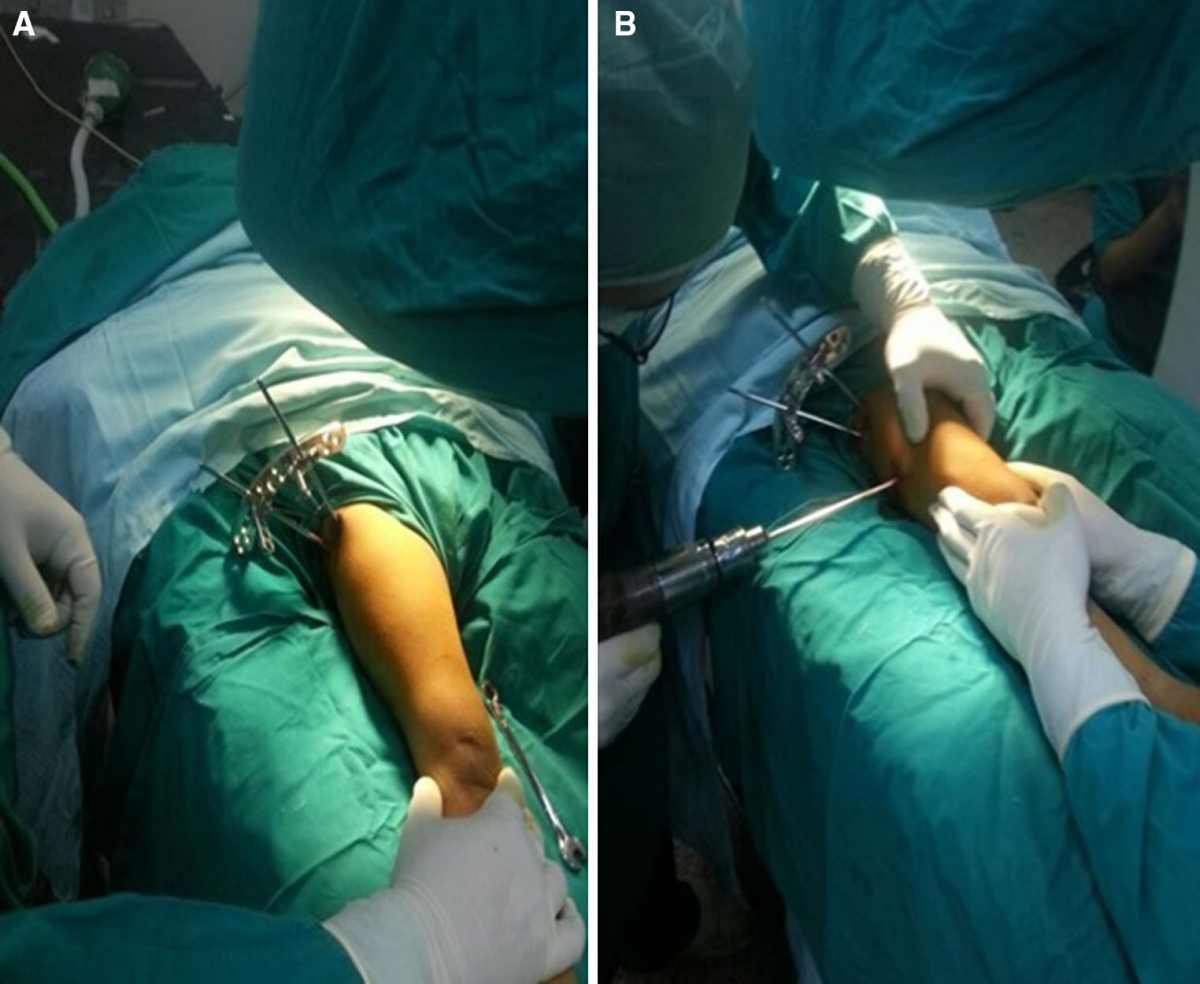
**a** Knee neutral position; **b** first distal half pin introduced

**Fig. 4:**
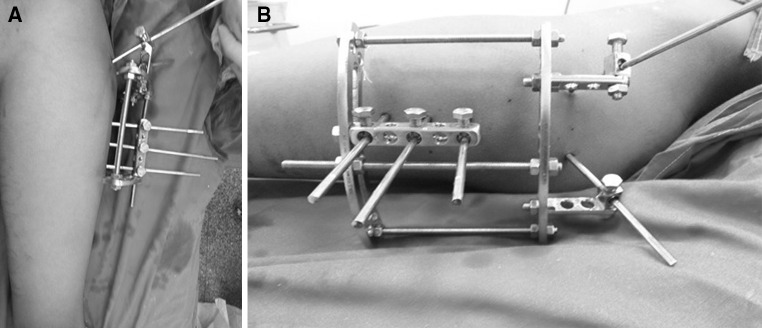
Proximal and distal arches are connected by 3 threaded rods, frontal (**a**) and side (**b**) views of the frame

Between January 2012 and January 2014, fourteen children (14 hips, 10 boys and 4 girls) with an average age of 6.4 years (range 3.8–11.5 years) sustained closed subtrochanteric femoral fractures and were treated operatively at Cairo University Hospital. Injury was caused by a fall from a height in 2 patients and through motor vehicle incidents in 12 patients. Two patients had abdominal injuries, and the remaining had isolated subtrochanteric femoral fractures. Radiographs revealed the most common fracture pattern to be a transverse fracture (in 8 patients); 3 had a spiral pattern, 2 patients a short oblique fracture, and one a fracture with a butterfly fragment. Surgery was performed 2–5 days after injury under general anaesthesia in the supine position and under fluoroscopic control. With leg internally rotated (hip neutral) (Fig. 1a), one half pin was inserted from anterolateral to posteromedial starting at the base of greater trochanter aiming at the lesser trochanter parallel to hip joint orientation line (line between tip of greater trochanter and femoral head centre, Fig. 1b). Using this pin to joystick the proximal fragment to correct flexion and external rotation, another half pin was inserted from posterolateral to anteromedial at a 90° angle to the first pin in the axial plane and parallel to hip joint orientation line (Fig. 2). With patella forward (knee neutral, Fig. 3a), three half pins were inserted perpendicular to the shaft (Fig. 3b) in the distal segment of the fracture with another half pin added in another plane in some cases. Proximal and distal pins were secured to Ilizarov arches that were then connected to one another by three threaded rods that were used for further improvement of the reduction in frontal plane (using the lateral rod) or in the sagittal plane (using the anterior and posterior rods, Fig. 4). Weight bearing was allowed as tolerated after surgery, and patients were followed up until union. The fixator was removed after complete union with no need for a spica cast or brace. The average follow-up was 18 months (range 12–24 months).

## Results

**Fig. 5:**
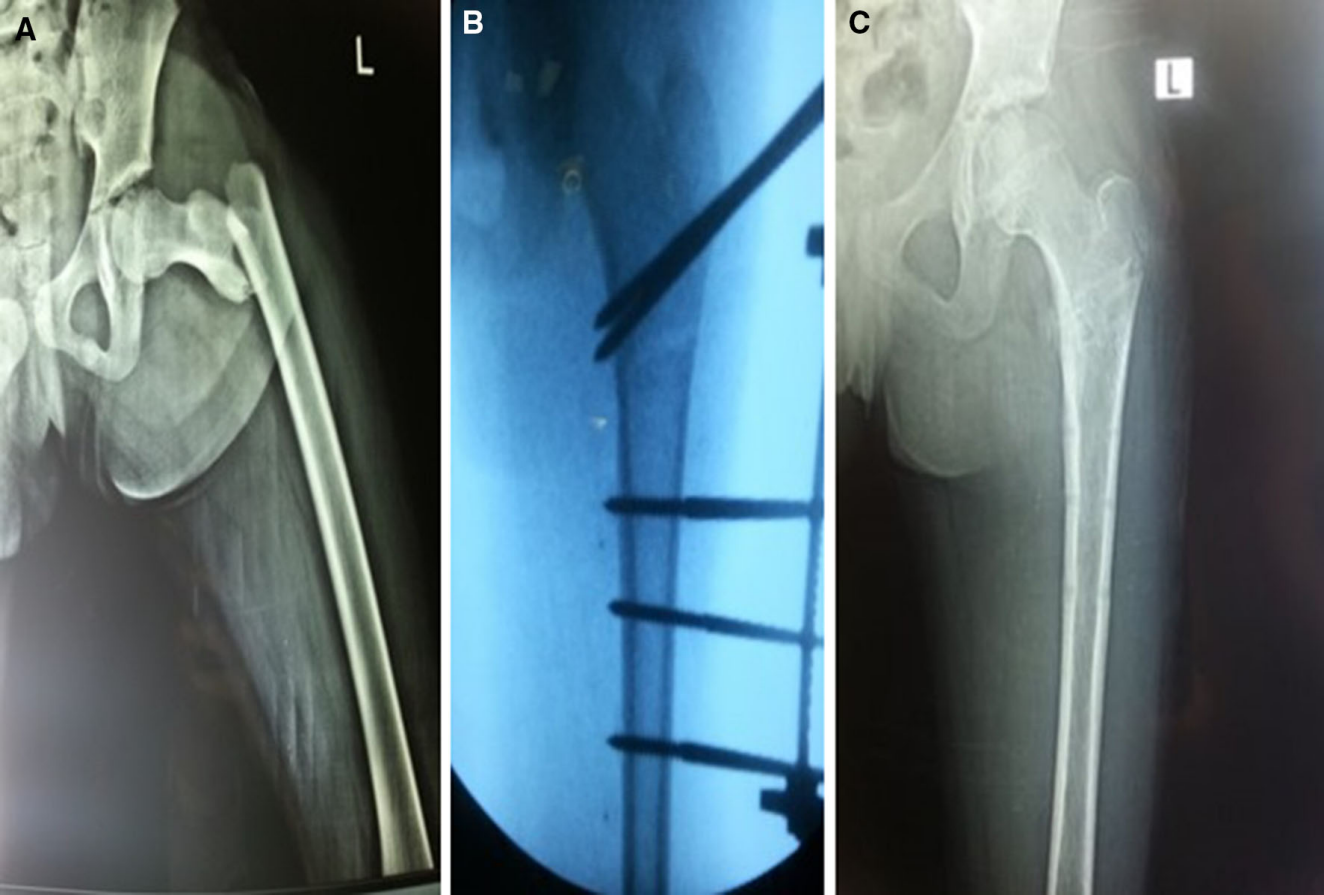
Preoperative AP X-rays (**a**) of 10-year-old female patient who sustained subtrochanteric fractures, intraoperative imaging after fixation (**b**). Anteroposterior (**c**) X-rays after complete union (11 weeks) and frame removal

All patients stayed in hospital for 2 days post-operatively except for those with abdominal injuries who were hospitalized for a longer period. All fractures united with anatomical alignment (Fig. 5) within an average of 8 weeks (range 6–12 weeks). Clinical evaluation at final follow-up revealed a full range of motion at both the hip and knee joints in all patients. There were no deep infections, but four patients had pin site infections that responded to oral antibiotics and pin site care. There were no incidences of refracture or avascular necrosis of the femoral head (Table 1).

**Table 1:** Patients data

	Age	Sex	Mechanism of injury	Complications	Fracture pattern	Time to union (week)
Case 1	6.4	M	Fall from height	–	Butterfly	8
Case 2	6.2	M	Motorcar accident	Pin site infection	Transverse	9
Case 3	7.4	F	Motorcar accident	–	Spiral	8
Case 4	6.1	M	Motorcar accident	–	Transverse	7
Case 5	3.8	M	Motorcar accident	–	Short oblique	6
Case 6	7	M	Motorcar accident	Pin site infection	Transverse	9
Case 7	7.3	M	Motorcar accident	–	Spiral	9
Case 8	7.1	F	Motorcar accident	–	Transverse	7
Case 9	7.6	M	Motorcar accident	Pin site infection	Transverse	9
Case 10	4.5	M	Motorcar accident	–	Short oblique	7
Case 11	4.1	F	Fall from height	–	Spiral	7
Case 12	4.7	M	Motorcar accident	–	Transverse	7
Case 13	6.4	M	Motorcar accident	–	Transverse	8
Case 14	11.5	M	Motorcar accident	Pin site infection	Transverse	12

## Discussion

Paediatric subtrochanteric femoral fractures are an unstable fracture type with little published in the literature [3]. Several studies have documented superior results with internal fixation compared to non-operative treatment [13]. According to Kregor et al. [10], the indications for operative fixation of paediatric femoral fractures were presence of an associated closed head injury and/or multiple injuries, open fractures and failure of conservative treatment. We applied the same indications for this study but extended the indications to include isolated subtrochanteric femoral fractures as we felt it difficult to maintain such fractures in an accepted position by non-operative means.

Aronson et al. [1] studied 54 children who had been treated with distal femoral 90/90 traction for an average of 24 days before being placed in a 1 1/2 hip spica cast. At an average follow-up of 4.3 years, all children were functionally normal and showed a symmetric range of motion of hip and knee. This method requires a long period of hospitalization and accurate control of fracture alignment with frequent radiographs and adjustments in traction as needed. The external fixation method proposed in this study requires less hospitalization time no major adjustment after application.

Hughes et al. [7] evaluated 23 children ranging in age from 2 through 10 years who had femoral fractures treated with early spica casting to determine the impact of treatment on the patients and their families. The greatest problems encountered by the family in caring for a child in a spica cast were transportation, cast intolerance by the child and hygiene. Although most children did not attend school while in the cast, no child was required to repeat a grade and the parents reported no permanent psychological effects. The researchers found treatment in a spica cast was much easier overall for families having preschool children rather than for those with school-age children. Such data should inform the decisions of orthopaedic surgeons and families who are trying to choose among the many options for young school-age children. In this study, transportation did not pose an issue as the child was allowed to weight-bear as tolerated and the after-care was easier.

Ferguson and Nicol [4] conducted a prospective study of early spica casting for children <10 years of age. They found that age >7 years was a variable predictive of a higher risk of failure of this technique for achieving satisfactory alignment. Martinez et al. [11] reported excessive shortening and angular deformity in 26 of 51 patients after immediate spica casting. In comparison, the small sample in this study showed an outcome without shortening or angular deformity for all ages treated with this configuration of Ilizarov external fixation.

Ward et al. [14] reported the use of a 4.5-mm AO dynamic compression plate for the treatment of femoral shaft fractures in 25 children, 6–16 years of age, 22 of whom had associated fractures or multisystem injury. The average time to fracture union was 11 weeks. Kregor et al. [10] reported on 12 patients who had 15 femoral fractures treated with compression plating with an average union time of 8 weeks. The average healing time in this study was comparable to that reported by Kregor et al.

Fyodorov et al. [6] reported hardware failure in 2 of 23 femoral fractures treated with dynamic compression plating. Hardware failure occurred at 6 weeks with one patient treated by revision plating and the other with spica casting; both fractures healed uneventfully. Ward et al. [14] reported one broken plate post-operatively in a boy who began full weight bearing a few days post-operatively. Implant failure did not occur in any patient in this series.

Good results have been reported using external fixators for femoral shaft fractures [2, 12], but the problem using external fixation in subtrochanteric femoral fractures was the limited room for application of the pins into the proximal femoral fragment. This problem is avoided by adopting the calcar fixation (rather than neck fixation) technique. There is better control of the proximal fragment, especially for correcting rotation and varus; this can be difficult to achieve or maintain with other minimally invasive internal fixation techniques, e.g. flexible intramedullary nails [5].

## Conclusions

We describe a new configuration of half pin insertion for subtrochanteric femoral fractures in children. Although the case series is of a small sample, the benefits have been shown to include avoidance of a large surgical exposure, decreased blood loss, risk of deep infection, an accurate and sustained reduction in the fracture displacement, early mobilization with a short hospital stay and avoiding the need for another open procedure for implant removal. Drawbacks include familiarity with the use of the Ilizarov fixator, the inconvenience to the patient of using the external fixator and the possibility of pin site-related problems.
